# Impact of Short-Chain Perfluoropropylene Oxide Acids on Biochemical and Behavioural Parameters in *Eisenia fetida* (Savigny, 1826)

**DOI:** 10.3390/jox15010002

**Published:** 2024-12-26

**Authors:** Davide Rotondo, Davide Gualandris, Candida Lorusso, Albert Braeuning, Antonio Calisi, Francesco Dondero

**Affiliations:** 1 Department of Science and Technological Innovation (DISIT), University of Eastern Piedmont, Viale Michel 11, 15121 Alessandria, Italy; davide.rotondo@uniupo.it (D.R.); davide.gualandris@uniupo.it (D.G.); candida.lorusso@uniupo.it (C.L.); antonio.calisi@uniupo.it (A.C.); 2 Department of Food Safety, German Federal Institute for Risk Assessment, Max-Dohrn-Str. 8-10, 10589 Berlin, Germany; albert.braeuning@bfr.bund.de

**Keywords:** acetylcholinesterase, catalase, immune response, phenol oxidase, poly and perfluoroalkyl substances, superoxide dismutase

## Abstract

Per- and polyfluoroalkyl substances (PFAS) are a class of persistent organic pollutants that pose a growing threat to environmental and human health. Soil acts as a long-term reservoir for PFAS, potentially impacting soil biodiversity and ecosystem function. Earthworms, as keystone species in soil ecosystems, are particularly vulnerable to PFAS exposure. In this study, we investigated the sublethal effects of three short-chain (C4–C6) next-generation perfluoropropylene oxide acids (PFPOAs) on the earthworm *Eisenia fetida*, using a legacy perfluoroalkyl carboxylic acid (PFCA), perfluorooctanoic acid (PFOA), as a reference. We assessed a suite of biochemical endpoints, including markers for oxidative stress (catalase and superoxide dismutase activity), immunity (phenol oxidase activity), neurotoxicity (acetylcholinesterase activity), and behavioural endpoints (escape test). Results indicate that all tested PFAS, even at sub-micromolar concentrations, elicited significant effects across multiple physiological domains. Interestingly, HFPO-DA demonstrated the most substantial impact across all endpoints tested, indicating broad and significant biochemical and neurotoxic effects. Our findings underscore the potential risks of both legacy and emerging PFAS to soil ecosystems, emphasising the need for further research to understand the long-term consequences of PFAS contamination.

## 1. Introduction

Per- and polyfluoroalkyl substances (PFAS) are persistent organic pollutants that have become a global health and environmental concern [1]. These chemicals, used in various industrial and consumer products, are ubiquitous in the environment, detected in air, water, soil, and biota [2,3]. Legacy PFAS, such as perfluorooctanoic acid (PFOA) and perfluorooctanesulfonic acid (PFOS), have been widely used for decades and are now subject to increased regulatory restrictions due to their persistent and bioaccumulative nature. In contrast, emerging PFAS are newer compounds developed to replace legacy PFAS and are often under-researched in terms of their environmental and health impacts. Major sources of PFAS release include industrial processes, firefighting foams, landfills, and wastewater treatment plants [4]. Due to their persistence and mobility, PFAS can accumulate in organisms and biomagnify through food webs [5,6].

Soil acts as a significant reservoir for PFAS [7]. The bioavailability of PFAS in soil depends on various factors, including soil properties, PFAS chain length, and the presence of other contaminants [8,9,10,11]. Longer-chain PFAS tend to be more strongly adsorbed to soil particles, while shorter-chain PFAS may leach more easily into groundwater or volatilise into the air [12,13].

PFAS contamination may pose a risk to soil biodiversity and ecosystem function. Earthworms are particularly vulnerable as a keystone species in soil ecosystems. These organisms play crucial roles in nutrient cycling, organic matter decomposition, and soil structure maintenance [14]. The toxic effects of PFAS on earthworms can disrupt these ecological functions and potentially impact higher trophic levels.

While acute toxicity of PFAS in earthworms and mammals, including humans, is relatively low [15,16], recent studies have highlighted their potential for sublethal effects across a wide range of organisms [17]. The International Agency for Research on Cancer (IARC) recently classified PFOA, a legacy perfluoroalkyl carboxylic acid (PFCA), as a Group 1 carcinogen due to its long-term toxicity in humans [18]. This underscores the importance of investigating sublethal mechanisms and chronic exposure effects of both legacy and emerging PFAS. Earthworms are valuable model organisms for assessing PFAS ecotoxicology [5,15,19,20]. They can readily accumulate a range of PFAS, including short-chain congeners, precursors, and fluorotelomers [6,19,21]. Biochemical and behavioural biomarkers in earthworms offer a sensitive tool for monitoring soil health and assessing the sublethal impacts of PFAS contamination [22,23,24,25].

In this study, we investigated the sublethal effects of PFAS on the earthworm *Eisenia fetida.* These included three perfluoropropylene oxide acids (PFPOAs) hexafluoropropylene oxide dimer acid (HFPO-DA, also known as GenX), perfluoro(2-methoxy)propanoic acid (PFMOBA), and perfluoro(3-methoxy)propanoic acid (PFMOPrA) along with perfluorooctanoic acid (PFOA) as a reference compound. HFPO-DA is widely used as a substitute for PFOA in the production of polytetrafluoroethylene (PTFE), and during this critical industrial process, PFMOBA and PFMOPrA may form as by-products [26]. Structurally, the three PFPOAs differ from each other by only one carbon in their chain length, enabling a comparative assessment of how specific alterations in molecular configuration influence their environmental behaviour and toxicity. By examining these closely related compounds, we aim to provide a deeper understanding of their modes of action, thereby informing risk assessments and guiding future regulatory decisions.

We exposed earthworms to PFAS through contact for 72 h and assessed a suite of biochemical endpoints (catalase, superoxide dismutase, phenol oxidase, and acetylcholinesterase activities) alongside behavioural responses, each reflecting distinct aspects of earthworm physiology. This research aims to enhance our understanding of the sublethal toxicity of emerging PFAS and contribute to a comprehensive risk assessment of these ubiquitous contaminants.

## 2. Materials and Methods

### 2.1. Chemicals

PFAS congeners of the highest available purity were purchased from the following suppliers:

2,2,3,3,4,4,5,5,6,6,7,7,8,8,8-pentadecafluorooctanoic acid (PFOA, CAS No. 335-67-1, 95%) from Merck (Darmstadt, Germany);

2,3,3,3-tetrafluoro-2-(1,1,2,2,3,3,3-heptafluoropropoxy)propanoic acid (HFPO-DA, CAS No. 13252-13-6, 97%) from SynQuest Laboratories, Inc. (Alachua, FL, USA);

2,2,3,3-tetrafluoro-3-(trifluoromethoxy)propanoic acid (PFMOPrA, CAS No. 377-73-1, 98%) from SynQuest Laboratories, Inc. (Alachua, FL, USA); and

2,2,3,3,4,4-hexafluoro-4-(trifluoromethoxy)butanoic acid (PFMOBA, CAS No. 863090-89-5, 97%) from Apollo Scientific (Bredbury, UK).

All other chemicals were of analytical grade.

### 2.2. Animals and Treatments

*Eisenia fetida* (Savigny, 1826) earthworms were cultured in a controlled environment (20 ± 2 °C, complete darkness) using a 1:1 mixture of sphagnum peat and organic garden soil, moistened to approximately 50% of its maximum water-holding capacity with de-ionised water. The pH was maintained at 6.0 ± 1.0 using calcium carbonate (CaCO_3_). Earthworms were fed weekly with manure from healthy, medication-free horses sourced from a local farm. A homogenous culture of adult earthworms (wet weight after gut content clearance: 0.58 ± 0.08 g, mean ± standard error) was obtained for the experiments. Adult earthworms were allowed to lay cocoons in fresh substrate for 4 weeks and then removed. The resulting juveniles were reared to adulthood (indicated by the appearance of a visible clitellum) for approximately 16 weeks.

Individual adult earthworms were placed in 7 cm plastic Petri dishes lined with a 3M No. 3 filter paper moistened with 1 mL of the respective PFAS solution or vehicle control (0.005% 2-propanol). Exposures were conducted for 72 h according to a modified OECD Test Guideline No. 207 filter paper contact test [27]. Stock solutions of PFOA, HFPO-DA, PFMOBA, and PFMOPrA were prepared in 2-propanol at 0.54 M [28] and then diluted in a 171 mOsmol kg^−1^ physiological solution, pH 7.3 [29], to achieve the desired test concentrations (229 μM, 31 μM, 4.2 μM, 0.6 μM). The concentration of 2-propanol in the control was minimised to match the concentration used in the test solutions.

The PFAS concentration range was determined through preliminary mortality tests conducted in accordance with OECD 207 guidelines, which confirmed that the tested PFAS congeners were non-lethal at concentrations up to the millimolar range. Sublethal concentrations were selected in the micromolar range to reflect levels reported in environments contaminated by AFFF-derived PFAS. The geometric progression, with a factor of e^2^ (where e is Euler’s number, approximately 2.718, and e^2^ ≈ 7.389), was initiated from environmentally measured levels of total PFAS found in soil at a pilot site studied within the framework of the SCENARIOS project (https://scenarios-project.eu) (accessed on 1 November 2024), ensuring an even distribution across the range while maintaining environmental relevance. Five experimental groups were established for each PFAS, with each group exposed to a different concentration. Each group consisted of 10 replicates (Petri dishes), with one earthworm per dish. Exposures were conducted at 20 °C in the darkness, with humidity controlled to prevent filter paper desiccation. Following the 72h exposure, earthworms were immediately subjected to a behavioural escape test (see Section 2.6) and then stored at −80 ± 1 °C until further analysis.

### 2.3. Biochemical Determinations

#### Oxidative Stress Biomarkers

Earthworms were homogenised in an ice-cold buffer (100 mM Tris-HCl, pH 7.6, 0.1% Triton X-100) using a glass–Teflon Potter–Elvehjem homogeniser. Homogenates were centrifuged at 10,000× *g* for 20 min at 4 °C, and the resulting supernatant (SN10) was collected. Catalase (CAT) activity in SN10 was determined using a native in-gel assay [30]. Total protein content in SN10 was determined using the Bradford assay with bovine serum albumin as a standard. Briefly, SN10 samples were diluted to a final protein concentration of 7.5 μg per sample in 0.05 M Tris-HCl, pH 6.8, 50% glycerol, 0.05% bromophenol blue. Samples and bovine erythrocytes catalase (1 μg) were loaded onto precast non-denaturing 12% polyacrylamide gels (Mini-PROTEAN^®^ TGX™, Bio-Rad Laboratories, Hercules, CA, USA) and electrophoresed at 150 V for 90 min at 4 °C in running buffer, 0.05 M Tris-HCl, pH 8.3, 0.3 M glycine.

After electrophoresis, gels were washed three times for 5 min in ultrapure water (18 MΩ·cm) and then incubated for 10 min in a freshly prepared 0.01% hydrogen peroxide solution diluted from a 30% stock (Merck, Darmstadt, Germany). Gels were washed again twice in ultrapure water and then incubated with gentle agitation in a freshly prepared staining solution containing 2% ferric chloride hexahydrate and 2% potassium ferricyanide trihydrate (Merck, Darmstadt, Germany). Catalase activity appeared as clear bands against a blue-green background. Band intensities were semi-quantified by densitometry using ImageJ v.1.54K software and compared to a standard curve generated with bovine catalase.

Superoxide dismutase (SOD) activity was also determined in SN10 using a competitive assay. This assay, based on the inhibition of tetrazolium salt reduction by superoxide radicals, was performed using a commercial reagent system (SOD Assay Kit, Merck, Darmstadt, Germany) and an SOD standard curve for quantification. Each sample in the 96-well plate contained 7.5 µg of protein in a final volume of 100 µL.

### 2.4. Phenol Oxidase Activity

Phenol oxidase (PO) activity was determined in the SN10 supernatant. The assay was performed in 96-well microplates using a reaction mixture containing 100 mM Tris-HCl buffer, pH 8.0, 50 mM CaCl_2_, and 10 mM L-DOPA, as reported by Prochazkova et al. (2019) [31]. Eighty microliters (80 µL) of this reaction mixture were combined with 7.5 μg of protein in 10 µL of each SN10 sample. The microplate was then incubated for 4 h at 25 °C in a dark environment. Following incubation, the absorbance at 492 nm was measured using an Infinite F200 PRO spectrophotometer (Tecan Group Ltd., Männedorf, Switzerland).

### 2.5. Acetylcholinesterase Activity

Acetylcholinesterase (AChE) activity was determined spectrophotometrically in the SN10 supernatant using a commercially available kit (Acetylcholinesterase Assay Kit, Ikzus, Italy) adapted to a microplate format. Briefly, 7.5 µg of protein in a final volume of 200 µL was added to each well. The assay was based on the conversion of acetylthiocholine to thiocholine by AChE, which subsequently reacts with Ellman’s reagent (5,5′-dithiobis-(2-nitrobenzoic acid), DTNB) to produce a yellow product. The reaction kinetics were monitored at 405 nm for 10 min using a microplate reader, and the AChE activity was calculated as nmol/min/mg protein after pathlength correction.

### 2.6. Escape Test

The escape test was conducted in a circular plastic arena (approximately 40 cm in diameter) containing an inverted 9 cm Petri dish lid placed in the centre. One millilitre (1 mL) of 200 mM NaCl solution was deposited onto the centre of the lid. An individual earthworm was then placed directly into the NaCl droplet, and the time taken for the earthworm to completely leave the droplet was recorded. A maximum escape time of 20 min was set; if an earthworm did not exit within this time frame, the escape time was recorded as 20 min. This escape test protocol was a modification of the method described by Da Cruz Jung et al. (2021) [32]. After each trial, both the arena and Petri dish lid were thoroughly rinsed with tap water, followed by distilled water to remove any residual mucus that could potentially influence subsequent tests.

### 2.7. Statistical Analysis

Statistical analysis of the data was performed using GraphPad Prism™ 9 (GraphPad Software, San Diego, CA, USA) and Systat 12 (Systat Software Inc., San Jose, CA, USA). Data were first assessed for normality and homoscedasticity. Normally distributed data with equal variances were analysed using one-way analysis of variance (ANOVA), followed by Holm–Sidak’s multiple comparison test. In cases of unequal variances, Brown–Forsythe ANOVA with Dunnett’s T3 post hoc test was applied. For data that did not meet the assumptions of normality, the non-parametric Kruskal–Wallis test was used, followed by either Dunn’s multiple comparison test or an uncorrected Dunn’s multiple comparison test.

## 3. Results

### 3.1. Antioxidant Enzyme

#### 3.1.1. Catalase Activity

**Figure 1:**
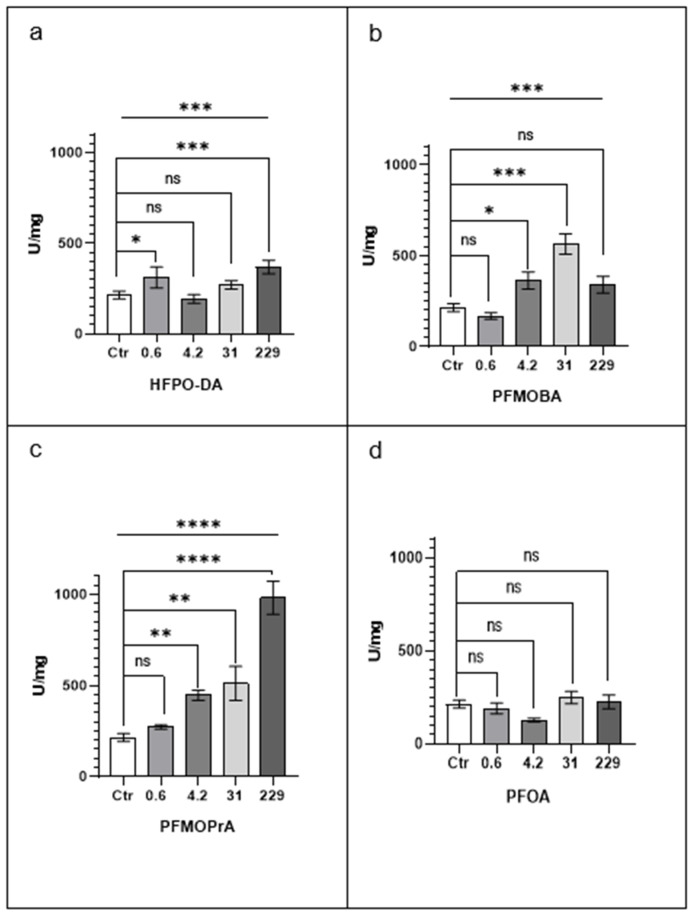
CAT (catalase) activity. Data are presented as the mean activity per mg of protein ± standard error of the mean (SEM). Statistical significance was determined by the Kruskal–Wallis test followed by a post hoc Dunn’s multiple comparison test. * *p* < 0.05; ** *p* < 0.01; *** *p* < 0.001; **** *p* < 0.0001; ns, not statistically significant. The x-axis reports PFAS concentrations in μM, and Ctr represents vehicle-exposed earthworms. Lines above the histogram bars indicate statistical significance between PFAS-treated groups and the control. Each panel represents results for a different PFAS compound: (**a**) HFPO-DA, (**b**) PFMOBA, (**c**) PFMOPrA, and (**d**) PFOA.

The effects of a 72 h exposure to PFOA and three PFPOA congeners—HFPO-DA, PFMOBA, and PFMOPrA—on the catalase (CAT) activity of *Eisenia fetida* tissue supernatants are presented in Figure 1.

Overall, exposure to PFPOA congeners resulted in significant increases in catalase activity compared to the control, whereas PFOA did not induce a consistent trend. Among the PFPOAs, PFMOPrA exhibited the most pronounced and concentration-dependent effect.

At 0.6 µM, only HFPO-DA caused a significant increase in catalase activity compared to the control (*p* < 0.05). PFMOBA, PFMOPrA, and PFOA did not show significant changes at this concentration. At 4.2 µM, both PFMOBA and PFMOPrA significantly increased catalase activity (*p* < 0.05 and *p* < 0.01, respectively), while HFPO-DA and PFOA did not exhibit significant effects. PFMOPrA’s effect was more substantial than that of PFMOBA at this concentration. At 31 µM, PFMOBA and PFMOPrA continued to show significant enhancements in catalase activity (*p* < 0.001 and *p* < 0.01, respectively). HFPO-DA did not elicit significant changes at this concentration, and PFOA remained without effect. At 229 µM, PFMOPrA induced the most pronounced increase in catalase activity, tripling enzyme functionality compared to the control (*p* < 0.0001). HFPO-DA also showed a significant increase at this concentration (*p* < 0.001), whereas PFMOBA did not produce a significant effect. PFOA did not significantly affect catalase activity at this concentration.

In contrast to the PFPOA congeners, PFOA exposure did not result in significant increases in catalase activity at any concentration. At 4.2 µM, PFOA exposure led to a decrease in catalase activity that approached statistical significance (*p* = 0.05).

Comparatively, PFMOPrA demonstrated the most consistent and robust increase in catalase activity across all concentrations, indicating a clear concentration-dependent effect. PFMOBA also showed significant increases at intermediate concentrations but lacked a consistent dose-response trend. HFPO-DA’s effects were significant only at the lowest and highest concentrations, without a clear pattern. The lack of effect by PFOA suggests a distinct mode of action compared to the PFPOA congeners.

These findings indicate that PFPOA congeners, particularly PFMOPrA, may induce oxidative stress responses in earthworms, as evidenced by increased catalase activity, whereas PFOA does not elicit a similar response.

#### 3.1.2. Superoxide Dismutase Activity

**Figure 2:**
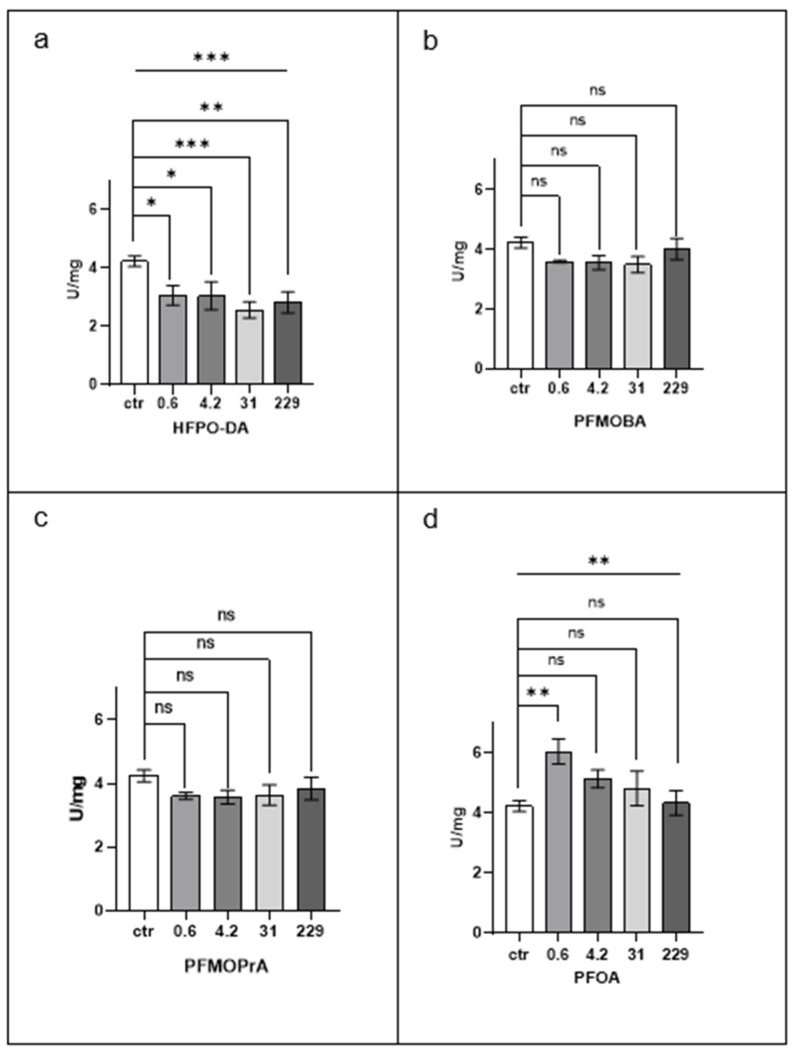
SOD (superoxide dismutase) activity. Statistical significance was determined by ANOVA followed by a post hoc Holm–Sidak’s multiple comparison test. * *p* < 0.05; ** *p* < 0.01; and *** *p* < 0.001; ns, not statistically significant. The line above the histogram bars indicates statistical significance between PFAS-treated groups and the control. Each panel represents results for a different PFAS compound: (**a**) HFPO-DA, (**b**) PFMOBA, (**c**) PFMOPrA, and (**d**) PFOA. See caption to Figure 1 for more details.

Figure 2 illustrates the effects of PFAS exposure on superoxide dismutase (SOD) activity in *Eisenia fetida* tissue extracts.

PFOA treatment resulted in an overall increase in SOD activity, primarily driven by a significant enhancement at the lowest concentration tested (0.6 µM, *p* < 0.001). No significant changes were, in fact, observed at higher concentrations.

HFPO-DA exposure caused a consistent and significant decrease in SOD activity across all concentrations. The most substantial reduction occurred at 31 µM (*p* < 0.001), with significant decreases to a similar extent also noted at 0.6 µM, 4.2 µM, and 229 µM (*p* < 0.05). This suggests that HFPO-DA inhibits SOD activity in a concentration-independent manner.

PFMOBA and PFMOPrA exposures did not produce statistically significant changes in SOD activity at any concentration, indicating a lack of effect on this enzyme.

Comparing the compounds, PFOA and HFPO-DA exhibited opposing effects on SOD activity. PFOA increased SOD activity at low concentrations, while HFPO-DA decreased it across all concentrations. PFMOBA and PFMOPrA did not significantly affect SOD activity, suggesting they do not impact this aspect of the antioxidant defence system.

These results imply that PFOA and HFPO-DA may modulate oxidative stress pathways differently, with PFOA potentially enhancing and HFPO-DA inhibiting the earthworm’s ability to mitigate oxidative stress through SOD activity.

### 3.2. Phenol Oxidase Activity

**Figure 3:**
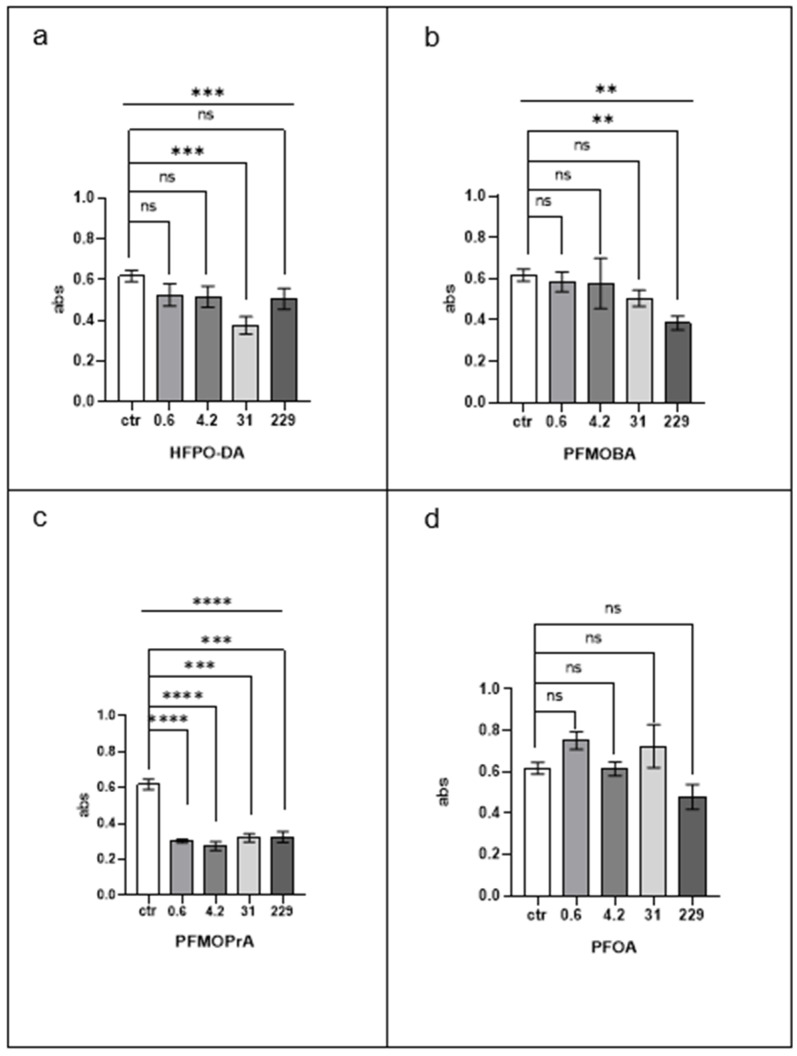
Phenol oxidase. A semi-quantitative evaluation was performed following the absorption (abs) at 590 nm due to L-DOPA oxidation. Statistical significance was determined by the Kruskal–Wallis test followed by a post hoc Dunn’s multiple comparison test. ** *p* < 0.01; *** *p* < 0.001; and **** *p* < 0.0001; ns, not statistically significant. The line above the histogram bars indicates statistical significance between PFAS-treated groups and the control. Each panel represents results for a different PFAS compound: (**a**) HFPO-DA, (**b**) PFMOBA, (**c**) PFMOPrA, and (**d**) PFOA. See caption to Figure 1 for more details.

Figure 3 presents the changes in phenol oxidase (PO) activity in the haemolymph (serum) of *Eisenia fetida* after exposure to PFAS.

HFPO-DA exposure resulted in a significant reduction in PO activity compared to control levels at all concentrations tested. The most pronounced decrease was observed at 31 µM (*p* < 0.001).

PFMOBA exposure led to a significant decrease in PO activity only at the highest concentration of 229 µM (*p* < 0.01), while lower concentrations did not produce significant effects.

PFMOPrA demonstrated the strongest inhibitory effect on PO activity among the compounds tested, with significant reductions observed at all concentrations (*p* < 0.0001). This indicates a potent effect on the earthworm’s immune function, as PO is crucial in defence mechanisms.

PFOA exposure did not significantly affect PO activity at any concentration, suggesting it does not impact this immune parameter.

PFMOPrA had the most substantial inhibitory effect on PO activity, followed by HFPO-DA. PFMOBA’s effect was significant only at the highest concentration, and PFOA did not affect PO activity.

These results suggest that the emerging PFPOA compounds, particularly PFMOPrA and HFPO-DA, may impair immune function in earthworms by inhibiting PO activity, whereas PFOA lacks this effect.

### 3.3. Acetylcholinesterase Activity

**Figure 4:**
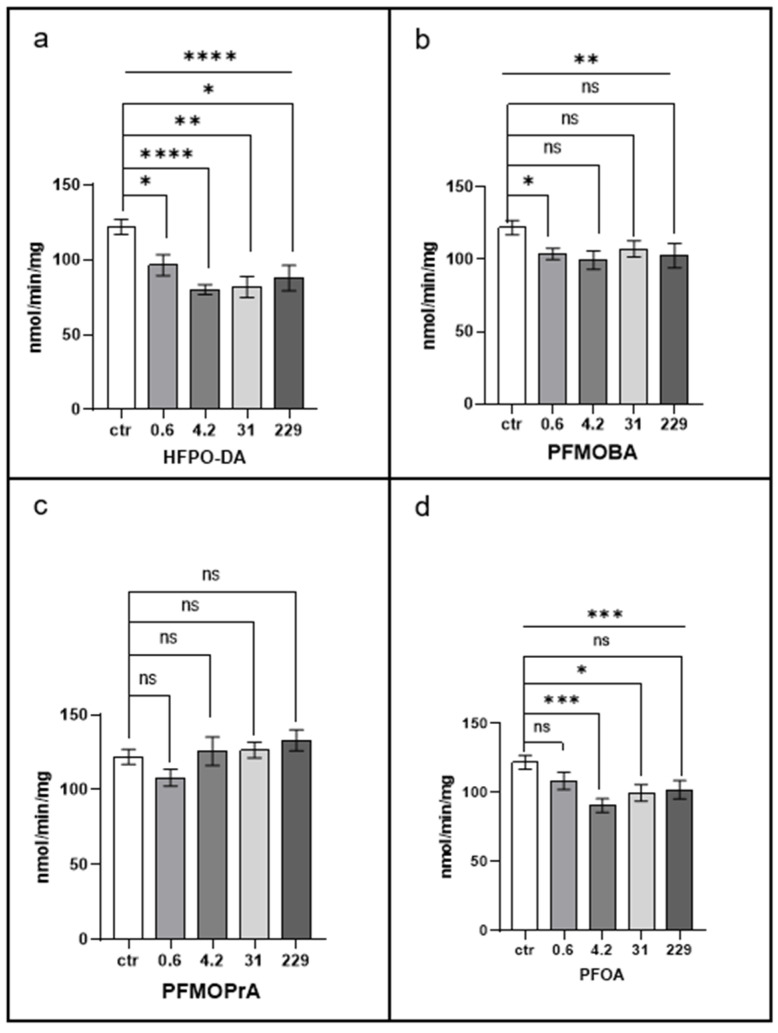
Acetylcholinesterase activity. Statistical significance was determined by the Brown–Forsythe ANOVA test, followed by a post hoc Dunnett’s T3 test. * *p* < 0.05; ** *p* < 0.01; *** *p* < 0.001; and **** *p* < 0.0001; ns, not statistically significant. The line above the histogram bars indicates statistical significance between PFAS-treated groups and the control. Each panel represents results for a different PFAS compound: (**a**) HFPO-DA, (**b**) PFMOBA, (**c**) PFMOPrA, and (**d**) PFOA. See caption to Figure 1 for more details.

Figure 4 depicts the effects of PFAS exposure on acetylcholinesterase (AChE) activity in *Eisenia fetida* tissues.

HFPO-DA exposure resulted in a significant decrease in AChE activity across multiple concentrations. Significant reductions were observed at 0.6 µM (*p* < 0.01), 4.2 µM (*p* < 0.001), 31 µM (*p* < 0.0001), and 229 µM (*p* < 0.05), indicating a dose-dependent inhibitory effect.

PFOA exposure led to a moderate decrease in AChE activity that was statistically significant at 4.2 µM (*p* < 0.05), suggesting a potential inhibitory effect, though less pronounced than that of HFPO-DA.

PFMOBA exposure exhibited threshold-like effects, with a significant reduction observed only at 229 µM (*p* < 0.05).

PFMOPrA did not significantly affect AChE activity at any concentration tested, indicating a lack of effect on this enzyme.

Comparing the compounds, HFPO-DA had the most robust inhibitory effect on AChE activity, showing significant decreases across all concentrations. PFOA and PFMOBA exhibited moderate inhibitory effects at specific concentrations, while PFMOPrA did not affect AChE activity.

These findings suggest that HFPO-DA may interfere with cholinergic neurotransmission in earthworms by inhibiting AChE activity, which could impact neuromuscular function.

### 3.4. Escape Test

**Figure 5:**
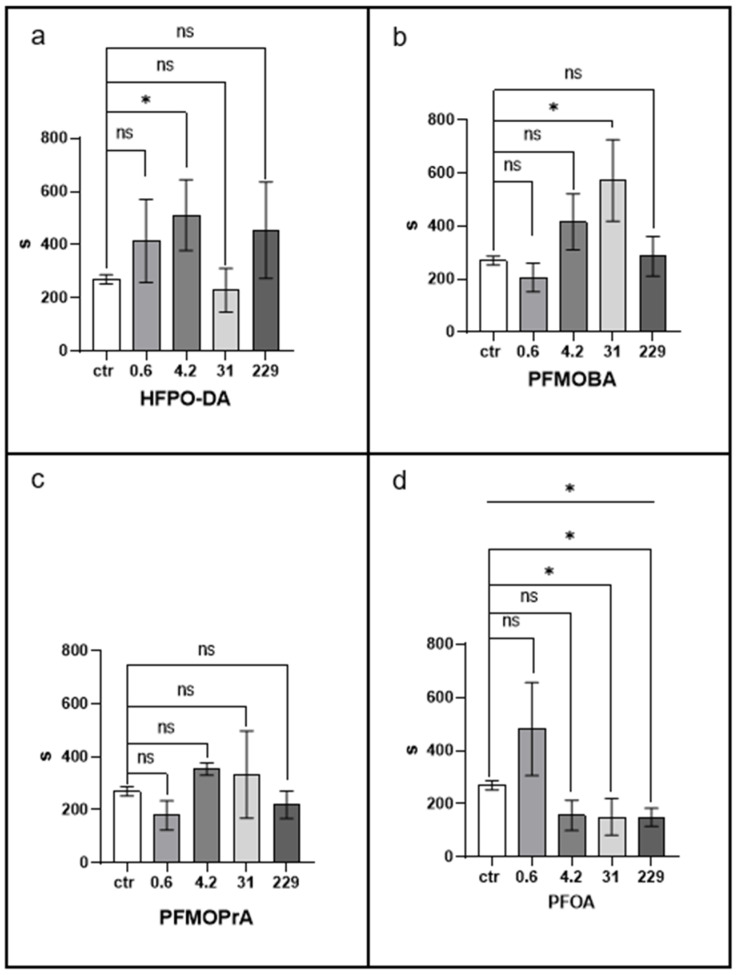
Escape test. Statistical significance was determined by the Kruskal–Wallis test, followed by a post hoc uncorrected Dunn’s test. * *p* < 0.05; ns, not statistically significant. The line above the histogram bars indicates statistical significance between PFAS-treated groups and the control. Each panel represents results for a different PFAS compound: (**a**) HFPO-DA, (**b**) PFMOBA, (**c**) PFMOPrA, and (**d**) PFOA. See caption to Figure 1 for more details.

The behavioural effects of PFAS exposure were evaluated using an escape latency test, with results presented in Figure 5.

PFOA exposure resulted in a significant reduction in median escape latency compared to the control group (*p* < 0.05), indicating a quicker escape response. This effect was more consistent across the higher concentrations tested.

HFPO-DA and PFMOBA exposures led to increases in escape latency times, with some specimens exhibiting prolonged responses. For HFPO-DA, several earthworms failed to escape within the full duration of the assay (20 min), resulting in a positively skewed distribution and higher mean latency times.

PFMOPrA exposure did not produce significant changes in median escape latency compared to the control, though the data distribution showed instances of both longer and shorter latency times.

The distribution of latency times for PFAS-exposed groups differed significantly from that of the control group (Kolmogorov–Smirnov test, *p* < 0.05), indicating that PFAS exposure affects the behavioural responses of earthworms (Table 1).

**Figure 6:**
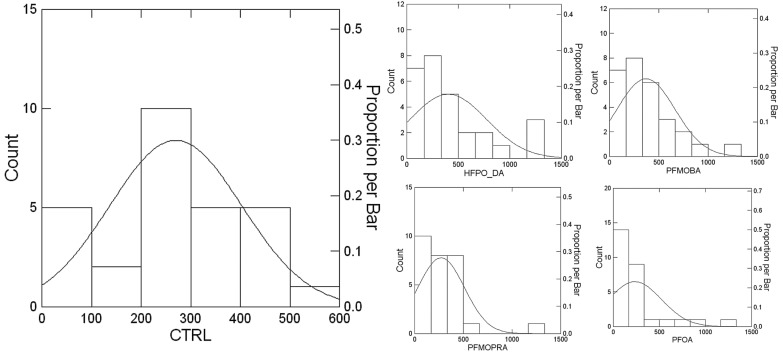
Histogram of latency times obtained from the escape test. The binned counts show the deviations of PFAS specimens from the symmetrical distribution of the control group. A biphasic response is observed, with a general increase in latency times for PFAS treatment. However, PFOA showed quicker median performance as judged by the U-statistics results (see body text).

Figure 6 shows histograms of latency times, highlighting deviations from the symmetrical distribution observed in the control group. PFAS-treated groups exhibited non-normal distributions with positive skewness, suggesting the presence of more extreme values.

PFOA exposure led to a faster escape response, while HFPO-DA and PFMOBA exposures resulted in delayed responses for some specimens. PFMOPrA had variable effects without significant changes in median latency.

These results suggest that PFAS compounds can affect the behavioural responses of earthworms, potentially impacting their ability to respond to adverse environmental conditions.

A Principal Component Analysis (PCA) was conducted using all data gathered in this study (Supplementary Figure S1). The PCA revealed separation among the treatments, suggesting distinct or partially overlapping modes of action and biochemical interactions for each PFAS congener. HFPO-DA and PFOA appeared clearly separated in the first and fourth quadrants of the loading plot, respectively. PFMOBA was intermediate, while PFMOPrA was separated in the third quadrant. The differences observed in the PCA were driven primarily by catalase and SOD activities in the first principal component (PC1) and acetylcholinesterase activity in the second principal component (PC2).

To complement the PCA analysis, an expert system classification was applied to compare the relative potency of the PFAS congeners based on five biochemical and behavioural endpoints: catalase, SOD, phenol oxidase, acetylcholinesterase activities, and escape latency. HFPO-DA ranked as the most potent compound, with the highest cumulative score (25), followed by PFMOPrA (20), PFMOBA (13), and PFOA (10). This classification, excluding PCA to avoid redundancy, highlighted HFPO-DA’s consistent and broad toxicity profile, underscoring its significant ecotoxicological risk. Further details are provided in Supplementary Figure S2 and in Tables S1–S3 (see Supplementary Information).

## 4. Discussion

The accumulation of PFAS in the environment and their subsequent transfer through trophic chains have led to increased studies aimed at determining their impact on ecosystems. Ecotoxicological studies on well-characterised environmental models, such as earthworms, are valuable tools for assessing the potential toxicity of persistent contaminants like PFAS, focusing on their bioavailable fraction. Numerous studies report the accumulation of PFAS in various invertebrate organisms [6,8,33,34,35,36] and vertebrates [37,38,39,40]. The effects of PFAS on these organisms are diverse, varying according to their composition (with thousands of congeners), concentration, exposure time, and the species affected. Therefore, exposure to PFAS can lead to a range of consequences, from subtle biochemical changes to severe physiological disruptions.

One target of PFAS is cellular metabolism, and it has been observed that PFAS toxicity manifests at the molecular level by altering enzyme activities involved in metabolic pathways. This can affect growth, reproduction, and, in the most severe cases, the survival of the organism. In human hepatoma cells (HepG2), exposure to PFOA and PFOS resulted in an increase in the activity of superoxide dismutase (SOD) and catalase (CAT), as reported by Bonato et al. (2020) [41] in a review of findings on PFAS affecting oxidative stress biomarkers in vitro and in vivo. These studies showed that PFOA and PFOS can overwhelm the balance of the antioxidant system, increase ROS generation, affect mitochondria, and initiate apoptosis. Mitochondrial dysfunction has also been reported in zebrafish [42].

Our study provides critical insights into the biochemical and behavioural impacts of per- and polyfluoroalkyl substances (PFAS) on terrestrial oligochaetes, particularly *Eisenia fetida*. Given the limited data on PFAS effects in terrestrial organisms, our aim was to evaluate a broad spectrum of biochemical biomarkers, including parameters related to oxidative stress, immunotoxicity, and neurotoxicity, with a particular focus on behaviour. In our study, we found no mortality up to a concentration of 4.6 mM suggesting that acute toxicity is not the primary concern with PFAS, including next-generation congeners. This finding is consistent with previous findings with either legacy PFAS, i.e., no mortality up to 100 mg kg^−1^ [43], or alternative congeners such as C6O4, a perfluoroalkyl ether compound [16]. In the latter case, significant acute toxicity is reported only at high concentrations (NOEC, 1390 mg/kg dry weight (d.w.)) while highlighting more pronounced reproductive effects at lower levels (EC50: 10.4 mg/kg d.w.; EC10: 0.8 mg/kg d.w.). These results emphasise the potential for chronic toxicity due to sublethal impacts observed at sub-micromolar concentrations.

The first hypothesis of PFAS toxicity we tested was the superoxide theory, which can lead to mitochondrial dysfunction and neurotoxicity as shown in nematodes [44]. In our study, we observed a significant response of superoxide dismutase (SOD) activity in organisms exposed to PFOA, characterised by an initial increase at lower exposure levels followed by a return to basal levels. This pattern mirrors findings reported by Wang et al. (2021) [45] in terrestrial oligochaetes, where SOD activation was noted at low pollutant levels but declined at higher PFOA concentrations. Interestingly, our study also revealed a similar trend in earthworms exposed to HFPO-DA, with all exposure conditions (ranging from 0.6 to 229 μM) resulting in SOD inhibition. Additionally, while PFMOBA and PFMOPrA induced slight and non-significant alterations in superoxide dismutase activity in *Eisenia fetida*, these changes indicated a trend towards reduced enzyme function. The exact mechanisms behind these effects are difficult to pinpoint, but some authors have suggested the possibility of enzyme inhibition due to the accumulation of oxidising agents in the organism’s body [45]. Moreover, it has been shown that certain PFAS congeners, such as PFOA and PFOS, can bind to the SOD enzyme, causing a change in its function [46,47]. The antioxidant defence system plays a crucial role in the toxicological interactions of PFOS and PFOA in *Daphnia magna* [46]. Exposure to PFAS appears to promote the accumulation of superoxide anions, which may induce the activation of the SOD synthesis pathway through mechanisms such as gene expression changes. The observed reduction in activity may also reflect a compensatory response following an initial enzyme peak or a metabolic effect at the level of gene or protein expression [48]. These factors could not be fully tested due to the limitations of our experimental design, which prioritised the number and concentrations of PFAS congeners in alignment with the 72 h period specified in OECD Test Guideline No. 207. However, the observed loss of SOD activity may indicate long-term toxicity of PFAS and therefore warrants further investigation in future studies.

The catalytic action of SOD is generally followed by catalase activity, which completes the detoxification process eliminating hydrogen peroxide from the cytoplasm. The various tests carried out in this study showed different responses of catalase between the legacy PFAS (PFOA) and the PFPOA congeners. In fact, the interesting aspect is that what has been observed with SOD is reciprocal to the effects on catalase: PFOA had no significant effects, whereas HFPO-DA, PFMOBA, and PFMOPrA appeared to stimulate catalase activity in *Eisenia fetida* in an increasing manner with C4 > C5 > C6. This means that these substances are likely to cause an increase in ROS concentration and a proactive detoxifying response. PFMOPrA induced a clear concentration-dependent increase in the detoxifying activity of CAT. In PFMOBA, this trend is also present with a decrease at the highest concentration, which is, however, an effect commonly described in the literature as the peak effect [49]. In fact, at the highest PFAS concentration (229 μM), excessive production of H_2_O_2_ might impair the antioxidant capacity of terrestrial oligochaetes, and, consequently, catalase was inhibited. Similar results have been reported by Zhao et al. (2017) [50], even though our study is based on a short-term filter paper exposure and theirs on a long-term approach in soil.

We then investigated the potential effects of new-generation PFAS compounds on the activity of phenol oxidase (PO), a key enzyme in the innate immune system. PO is crucial in the melanin cascade, promoting pathogen encapsulation and destruction. It is worth noting that there is a connection between superoxide and phenol oxidase activity. Both legacy and next-generation PFAS can alter the activity of antioxidant enzymes such as superoxide dismutase, catalase and peroxidase [41,51], which play an essential role in limiting the excessive formation of ROS by acting as a first line of defence against ROS-mediated tissue damage. Earthworm coelomocytes can synthesise melanin through the action of phenol oxidase, which is useful for encapsulating and dissociating pathogens from the organism. It should also be noted that highly reactive, toxic quinone intermediates are formed during this melanisation process [52]. Studies have shown that phenol oxidase has a fungicidal, bactericidal, and antiviral effect and is, therefore, an important component of the innate immune system of invertebrates [53]. In our study, a decrease in phenol oxidase activity was observed in PFAS-treated samples after exposure of earthworms to PFAS. The results of our investigation are consistent with previous studies that have demonstrated the detrimental effects of PFAS exposure on the activity of important enzymes involved in antioxidant defence mechanisms and innate immunity. The observed reduction in phenol oxidase activity indicates a potential disruption of the earthworms’ ability to mount an effective immune response against pathogens. In mammals, PFAS exposure has been shown to alter CD4+ and CD8+ T cell populations, reducing their functionality and increasing pro-inflammatory cytokines like IL-1β, which compromises cell-mediated immunity [54]. Moreover, over the past decade, numerous epidemiological studies have highlighted the ability of PFASs to affect the immune system through immunosuppressive action, finding a decrease in antibody production with increasing PFAS levels in serum [55,56]. Our findings of disrupted PO activity in earthworms suggest a potential commonality in PFAS-induced immunotoxicity across different immune systems. This highlights the need for further studies to explore these parallels and inform comprehensive risk assessments of PFAS exposure.

AChE plays a very important role in the function of the neuromuscular system as it prevents continuous muscle contractions. Inhibition of AChE causes dysfunction in organisms, such as behavioural changes, paralysis, and death. AChE activity has been used to analyse soil contamination effects mediated by a broad spectrum of molecules, such as organophosphate pesticides, carbamates, persistent organic pollutants (POPs), and polycyclic aromatic hydrocarbons (PAHs) [22,57,58]. Mulkiewicz et al. (2007) [59] demonstrated AChE inhibitory effects of PFAS, i.e., perfluorocarboxylic acid congeners, including PFOA with IC50 in the mM range using an *in chemico* assay with the eel AChE enzyme. A study by Li (2007) [60] in *Dugesia planaria* showed in vivo AChE inhibition (about 30%) at PFOA concentrations similar to the highest dose levels adopted in our study. Other studies in planaria and Daphnia reported enzyme modulation due to PFOS [61]. A study by Nusair et al. (2017) [62] on *Eisenia andrei* showed that polychlorinated flame retardants may have the ability to block the action of AChE in earthworm specimens by 30%, but no effects of PFAS on oligochaete have been reported so far. Nevertheless, Yang et al. (2019) [46] proposed a mechanism of inhibition due to physical interactions of PFAS and the enzyme molecule, as shown by docking studies using genuine *D. magna* AChE-inferred protein sequence. In our study, the effect found in tests conducted on HFPO-DA is very similar to that of PFOA. In contrast, PFMOPrA and PFMOBA show results that are of little or no statistical significance. The signalling mechanisms for the inhibition of AChE by PFAS are still unclear and under investigation. For other compounds, however, they appear to be based on regulation at the gene expression level. The fact that the differences in AChE activity were not extreme suggests that these chemicals do not have a specific effect on the catalytic site of the enzyme. However, the effects observed on HFPO-DA and PFOA suggest that these chemicals do influence the activity of AChE.

Previous work has shown that enzymatic inhibition of AChE and increased acetylcholine synthesis also affect behaviour [62]. In a recent study on *Eisenia fetida* exposed to polycyclic aromatic hydrocarbons such as fluorene, He et al. (2023) [63] reported how altered AChE signalling affects avoidance behaviour, i.e., the organisms tested are less efficient at perceiving the presence of abiotic stress and also show altered growth and metabolism. Moreover, studies on male mice exposed to PFOS during lactation showed alterations in locomotor behaviour; in fact, treated mice were less active at the beginning of the experiment and had high activity at the end [64]. These animals also showed a reduced transcription of the gene for AChE. Experiments concerning PFAS-induced neurotoxicity mostly involve vertebrate animals, while studies on invertebrates are very few, and one of these is the one carried out on *C. elegans* [65] subjected to PFAS treatment, in which the animals showed clear locomotor deficits. In our study, results show that PFAS exposure, including the relatively newer PFPOA congeners, exerts a possible neuromotor deregulation in *Eisenia fetida*, and this effect has often been associated with AChE inhibition, a common marker of neurotoxicity found to be affected by many organic compounds including dioxins [66]. Behavioural responses, assessed through the escape test [34], revealed statistically significant effects of PFOA on escape latency, with notable deviations from the control group. This observation aligns with the findings in *Lumbriculus variegatus*, where PFOS exposure significantly affected escape behaviour [67]. The data distribution for the propylene oxide acids showed significant deviations from the control group, with increased latency times suggesting neuromotor deregulation. These behavioural alterations might be linked to the neurotoxic effects observed through decreased AChE activity. Mechanistic studies on the (neuro)toxicity of new-generation PFASs such as HFPO-DA are still scarce in the literature. To our knowledge, the only data found in the literature are from an experiment conducted on zebrafish larvae exposed to HFPO-DA treatment from day 0 to day 5 post-fertilisation [68], where HFPO-DA did not elicit neurotoxicity during the developmental stages of this organism. Despite this negative evidence, further measuring of neurotransmitter levels in earthworm tissues might provide further clues about the observed effects on locomotion.

This study sheds light on the sublethal impacts of short-chain PFPOA congeners, which are currently under-investigated, on terrestrial invertebrates. This research reveals that the sublethal effects of these compounds are evident and correlate with their specific biochemical and behavioural profiles, as indicated by the comparative potency analysis. The results demonstrate that HFPO-DA (C6) exerts the most pronounced effects, followed by PFMOPrA (C4) and PFMOBA (C5), with PFOA showing the least impact. This highlights the importance of considering both molecular structure and functional endpoints when assessing the ecotoxicological risks of emerging PFAS compounds.

The hypothesis that HFPO-DA (C6) is the most active molecule among the tested PFAS is supported by its impact on a wide range of measured variables. HFPO-DA significantly increased catalase activity at both high (229 μM) and low (0.6 μM) concentrations, indicating a significant influence on oxidative stress response. It consistently decreased SOD activity across all concentrations, suggesting a disruption of the antioxidant defence system. Furthermore, HFPO-DA significantly reduced phenol oxidase activity at 31 μM, potentially impairing the immune response. Remarkably, it also showed the most substantial inhibitory effect on AChE activity among the tested PFAS, implying a pronounced delay time in the escape test. These findings, along with the PCA plot indicating a distinct separation of HFPO-DA from other congeners and the expert system classification (Supplementary Information), collectively suggest that HFPO-DA is the most potent disruptor of the measured biological parameters in *E. fetida*.

One possible explanation for HFPO-DA’s heightened potency is its unique hexafluoropropylene oxide functional group, which may enhance its ability to integrate into and disrupt biological membranes. This structural feature could facilitate interactions with key enzymes or proteins, leading to broader biochemical impacts. Additionally, HFPO-DA’s physicochemical properties, such as its optimal balance of hydrophobicity and hydrophilicity, may allow it to penetrate cell membranes more effectively, enabling greater access to intracellular targets. In contrast, PFMOPrA’s smaller size and simpler methoxy-functionalised structure may enhance its mobility and interaction with specific targets compared to PFMOBA, which is slightly larger and potentially less bioavailable. These structural differences provide a plausible basis for the observed ranking in toxicity and highlight the nuanced role of molecular structure in PFAS bioactivity.

This study bears limitations due to its short-term nature and would benefit from long-term investigations. However, it showed remarkable effects on vital aspects of earthworm metabolism, including antioxidant, immune, neurological, and behavioural functions, starting from concentrations that are commonly found in hotspots of PFAS contamination (0.6 μM PFOA equals nearly 250 ppb), such as firefighting sites and manufacturing districts. Our study reveals significant biochemical, immunotoxic, neurological, and behavioural effects of next-generation PFAS on earthworms at sub-micromolar levels. These findings highlight a potential threat to soil health and biodiversity. Earthworms play a crucial role in soil ecosystems by aiding nutrient cycling, organic matter decomposition, and maintaining soil structure. The observed adverse effects indicate that PFAS contamination can disrupt these essential ecosystem services, leading to broader ecological consequences. Disruption of earthworm physiological functions can impair soil aeration, nutrient cycling, and organic matter decomposition, potentially affecting soil fertility and plant growth. Moreover, earthworms serve as prey for a variety of animals, so adverse effects on earthworm populations could have cascading impacts on food webs.

By highlighting the sublethal effects of next-generation PFAS, our study underscores the importance of including terrestrial organisms in environmental risk assessments. Our results suggest that these compounds warrant closer scrutiny to understand their potential long-term impacts on soil ecosystems. Further research is needed to investigate the chronic toxicity of PFPOAs, including reproductive and developmental endpoints, and to explore the molecular mechanisms underlying the observed effects.

## 5. Conclusions

This study highlights the complex and varying impacts of different PFPOAs on terrestrial invertebrates, emphasising that while acute toxicity appears low, chronic exposure to even sublethal levels of these substances could result in significant physiological and behavioural disruptions. The observed effects on antioxidant enzymes, immune function, neurotoxicity markers, and behaviour underline the multifaceted impact of PFAS. Furthermore, the comparative potency analysis conducted in this study revealed that HFPO-DA exhibits the most pronounced effects, followed by PFMOPrA, PFMOBA, and PFOA, demonstrating the need for comprehensive ecotoxicological assessments that integrate biochemical and behavioural endpoints alongside systematic potency classification frameworks.

## Figures and Tables

**Table 1:** Escape latency time statistics. Shown are descriptive statistics obtained for the different tested conditions. All time data are expressed in seconds.

	CTRL	HFPO-DA	PFMOBA	PFMOPrA	PFOA
No. of cases	28	28	28	28	28
Minimum	22	15	8	10	2
Maximum	518	1220	1220	1220	1220
Range	496	1205	1212	1210	1218
Sum	7551	11,265	10,349	7591	6560
Median	283	21	296	257	169
Arithmetic Mean	269.68	402.35	369.61	271.11	234.29
SE	25.17	70.51	55.8	45.36	54.35
95,% Lower Confidence Limit	218.03	257.65	255.11	178.04	122.78
95,% Upper Confidence Limit	321.33	547	484.11	364.17	345.8
Standard Deviation	133.2	373.1	295.28	240.01	287.58
Shapiro–Wilk Statistics	0.96	0.84	0.91	0.79	0.74
Shapiro–Wilk *p*-value	0.26	0.001	0.016	0.0001	0.0001

## Data Availability

The original contributions presented in this study are included in the article. Further inquiries can be directed to the corresponding author.

## Supplementary Materials

The following supporting information can be downloaded at https://www.mdpi.com/article/10.3390/jox15010002/s1, Figure S1: Principal component analysis on standardised data; Figure S2: PFPOA potency vs. PFOA; Table S1: PFAS congener potency summary; Table S2: Scoring system; Table S3: Scoring system.

## References

[1] Pelch K.E., Reade A., Wolffe T.A.M., Kwiatkowski C.F. (2019). PFAS health effects database: Protocol for a systematic evidence map. Environ. Int..

[2] Houde M., Martin J.W., Letcher R.J., Solomon K.R., Muir D.C. (2006). Biological monitoring of polyfluoroalkyl substances: A review. Environ. Sci. Technol..

[3] Brase R.A., Mullin E.J., Spink D.C. (2021). Legacy and emerging per- and polyfluoroalkyl substances: Analytical techniques, environmental fate, and health effects. Int. J. Mol. Sci..

[4] Van Asselt E.D., Rietra R.P., Römkens P.F., van der Fels-Klerx H.J. (2011). Perfluorooctane sulphonate (PFOS) throughout the food production chain. Food Chem..

[5] Rich C.D., Blaine A.C., Hundal L., Higgins C.P. (2015). Bioaccumulation of perfluoroalkyl acids by earthworms (*Eisenia foetida*) exposed to contaminated soils. Environ. Sci. Technol..

[6] Burkhard L.P., Votava L.K. (2023). Biota-Sediment Accumulation Factors for Per- and Polyfluoroalkyl Substances. Environ. Toxicol. Chem..

[7] Brusseau M.L., Anderson R.H., Guo B. (2020). PFAS concentrations in soils: Background levels versus contaminated sites. Sci. Total Environ..

[8] Zhao S., Zhu L., Liu L., Liu Z., Zhang Y. (2013). Bioaccumulation of perfluoroalkyl carboxylates (PFCAs) and perfluoroalkane sulfonates (PFSAs) by earthworms (*Eisenia foetida*) in soil. Environ. Pollut..

[9] Zhao L., Zhu L., Zhao S., Ma X. (2016). Sequestration and bioavailability of perfluoroalkyl acids (PFAAs) in soils: Implications for their underestimated risk. Sci. Total. Environ..

[10] Ghisi R., Vamerali T., Manzetti S. (2019). Accumulation of perfluorinated alkyl substances (PFAS) in agricultural plants: A review. Environ. Res..

[11] Shahsavari E., Rouch D., Khudur L.S., Thomas D., Aburto-Medina A., Ball A.S. (2021). Challenges and current status of the biological treatment of pfas-contaminated soils. Front. Bioeng. Biotechnol..

[12] Li J., Sun J., Li P. (2022). Exposure routes, bioaccumulation and toxic effects of per- and polyfluoroalkyl substances (PFASs) on plants: A critical review. Environ. Int..

[13] Wang S., Liu T., Qian X., Wang H., Li M., Wang X., Wei S., Chen H. (2023). Microbial plankton responses to perfluoroalkyl acids and their alternatives in the aquatic environment. J. Hazard. Mat..

[14] Zhao S., Fang S., Zhu L., Liu L., Liu Z., Zhang Y. (2014). Mutual impacts of wheat (*Triticum aestivum* L.) and earthworms (*Eisenia fetida*) on the bioavailability of perfluoroalkyl substances (PFASs) in soil. Environ. Pollut..

[15] Karnjanapiboonwong A., Deb S.K., Subbiah S., Wang D., Anderson T.A. (2018). Perfluoroalkylsulfonic and carboxylic acids in earthworms (*Eisenia fetida*): Accumulation and effects results from spiked soils at PFAS concentrations bracketing environmental relevance. Chemosphere.

[16] Bizzotto E.C., Libralato G., Breda S., Siciliano A., Scanferla P., Vighi M., Marcomini A. (2024). Toxicity and bioaccumulation of the fluorosurfactant cC6O4 in the earthworm *Eisenia foetida* (Savigny, 1826). Sci. Total Environ..

[17] Calisi A., Baranzini N., Marcolli G., Bon C., Rotondo D., Gualandris D., Pulze L., Grimaldi A., Dondero F. (2024). Evaluation of per- and polyfluoroalkyl substances (PFAS) toxic effects on the acute inflammatory response in the medicinal leech Hirudo verbana. Chemosphere.

[18] Zahm S.H., Bonde J.P., Chiu W.A., Hoppin J.A., Kanno J., Abdallah M., Blystone C.R., Calkins M., Dong G., Dorman D.C. (2024). Carcinogenicity of perfluorooctanoic acid and perfluorooctanesulfonic acid. Lancet Oncol..

[19] Xu D., Li C., Wen Y., Liu W. (2013). Antioxidant defense system responses and DNA damage of earthworms exposed to Perfluorooctane sulfonate (PFOS). Environ. Pollut..

[20] Delor L., Louzon M., Pelosi C., Michel E., Maillet G., Carronnier H. (2023). Ecotoxicity of single and mixture of perfluoroalkyl substances (PFOS and PFOA) in soils to the earthworm *Aporrectodea caliginosa*. Environ. Pollut..

[21] Munoz G., Desrosiers M., Vetter L., Duy S.V., Jarjour J., Liu J., Sauvé S. (2020). Bioaccumulation of zwitterionic polyfluoroalkyl substances in earthworms exposed to aqueous Film-Forming foam impacted soils. Environ. Sci. Technol..

[22] Calisi A., Latino M.E., Corallo A., Grimaldi A., Ferronato C., Antisari L.V., Dondero F., Sanchez-Hernandez J.C. (2019). Biomarkers in Soil Organisms: Their Potential use in the Assessment of Soil Pollution and Remediation. Bioremediation of Agricultural Soils.

[23] Sanchez-Hernandez J.C. (2019). Bioremediation of Agricultural Soils.

[24] Xiao R., Ali A., Xu Y., Abdelrahman H., Li R., Lin Y., Bolan N., Shaheen S., Rinklebe J., Zhang Z. (2022). Earthworms as candidates for remediation of potentially toxic elements contaminated soils and mitigating the environmental and human health risks: A review. Environ. Int..

[25] Alves P.R.L., Bandeira F.O., Hennig T.B., Vig A.P., Singh J., Suthar S.S. (2022). Ecological Role of Earthworms as Bioindicators of Soil Health. Earthworm Engineering and Applications.

[26] Sun M., Arevalo E., Strynar M., Lindstrom A., Richardson M., Kearns B., Pickett A., Smith C., Knappe D.R.U. (2016). Legacy and emerging perfluoroalkyl substances are important drinking water contaminants in the Cape Fear River watershed of North Carolina. Environ. Sci. Technol. Lett..

[27] OECD (1984). Test No. 207: Earthworm, Acute Toxicity Tests, OECD Guidelines for the Testing of Chemicals, Section 2.

[28] Zhang C., McElroy A.C., Liberatore H.K., Alexander N.L.M., Knappe D.R.U. (2022). Stability of Per- and Polyfluoroalkyl Substances in Solvents Relevant to Environmental and Toxicological Analysis. Environ. Sci. Technol..

[29] Riedl S.a.B., Völkl M., Holzinger A., Jasinski J., Jérôme V., Scheibel T., Feldhaar H., Freitag R. (2021). In vitro cultivation of primary intestinal cells from Eisenia fetida as basis for ecotoxicological studies. Ecotoxicology.

[30] Lorusso C., Calisi A., Sarà G., Dondero F. (2022). In-gel assay to evaluate antioxidant enzyme response to silver nitrate and silver nanoparticles in marine bivalve tissues. Special Issue: Aquatic Toxicology and Aquaculture: Questions and Advances. Appl. Sci..

[31] Prochazkova P., Roubalova R., Skanta F., Dvorak J., Pacheco N.I.N., Kolarik M., Bilej M. (2019). Developmental and immune role of a novel multiple cysteine cluster tlr from *Eisenia andrei* earthworms. Front. Immunol..

[32] Da Cruz Jung I.E., Assmann C.E., Mastella M.H., Barbisan F., Spilliari Ruaro R.A., Roggia I., Osmarin Turra B., Chitolina B., de Oliveira Alves A., Teixeira C.F. (2021). Superoxide-anion triggers impairments of immune efficiency and stress response behaviors of *Eisenia fetida* earthworms. Chemosphere.

[33] Mommaerts V., Hagenaars A., Meyer J., De Coen W., Swevers L., Mosallanejad H., Smagghe G. (2011). Impact of a perfluorinated organic compound PFOS on the terrestrial pollinator *Bombus terrestris* (Insecta, Hymenoptera). Ecotoxicology.

[34] Marziali L., Rosignoli F., Valsecchi S., Polesello S., Stefani F. (2019). Effects of perfluoralkyl substances on a multigenerational scale: A case study with *Chironomus riparius* (diptera, chironomidae). Chemosphere.

[35] Sonter C.A., Rader R., Stevenson G., Stavert J.R., Wilson S.C. (2021). Biological and behavioural responses of European honey bee (*Apis mellifera*) colonies to perfluorooctane sulfonate exposure. Integr. Environ. Assess. Manag..

[36] Ma T., Ye C., Wang T., Li X., Luo Y. (2022). Toxicity of per- and polyfluoroalkyl substances to aquatic invertebrates, planktons, and microorganisms. Int. J. Environ. Res..

[37] Riebe R.A., Falk S., Georgii S., Brunn H., Failing K., Stahl T. (2016). Perfluoro-alkyl acid concentrations in livers of fox (*Vulpes vulpes*) and chamois (*Rupicapra rupicapra*) from Germany and Austria. Arch. Environ. Contam. Toxicol..

[38] Babut M., Labadie P., Simonnet-Laprade C., Munoz G., Roger M.-C., Ferrari BJ D., Budzinski H., Sivade E. (2017). Per- and poly-fluoroalkyl compounds in freshwater fish from the Rhône River: Influence of fish size, diet, prey contamination and biotransformation. Sci. Total Environ..

[39] Cleary R.S., Karnjanapiboonwong A., Thompson W.A., Lasee S.J., Subbiah S., Kaublé R.K., Andraski B.J., Anderson T.A. (2020). Emerging and Historical Con-taminants Detected in Desert Rodents Collected Near a Low-Level Radioactive Waste Site. Environ. Toxicol. Chem..

[40] Abercrombie S.A., de Perre C., Iacchetta M., Flynn R.W., Sepúlveda M.S., Lee L.S., Hoverman J.T. (2020). Sublethal Effects of Dermal Exposure to Poly- and Perfluoroalkyl Substances on Post metamorphic Amphibians. Environ. Toxicol. Chem..

[41] Bonato M., Corrà F., Bellio M., Guidolin L., Tallandini L., Irato P., Santovito G. (2020). PFAS Environmental Pollution and Antioxidant Responses: An Overview of the Impact on Human Field. Int. J. Environ. Res. Public Health.

[42] Ulhaq Z.S., Tse W.K. (2023). Perfluorohexanesulfonic acid (PFHxS) induces oxidative stress causes developmental toxicities in zebrafish embryos. J. Hazard. Mat..

[43] He W., Megharaj M., Naidu R. (2016). Toxicity of perfluorooctanoic acid towards earthworm and enzymatic activities in soil. Environ. Monit. Assess..

[44] Sammi S.R., Foguth R.M., Nieves C.S., De Perre C., Wipf P., McMurray C.T., Lee L.S., Cannon J.R. (2019). Perfluorooctane Sulfonate (PFOS) Produces Dopaminergic Neuropathology in Caenorhabditis elegans. Toxicol. Sci..

[45] Wang Z., Li C., Shao Y., Xue W., Wang N., Xu X., Zhang Z. (2021). Antioxidant defense system responses, lysosomal membrane stability and DNA damage in earthworms (*Eisenia fetida*) exposed to perfluorooctanoic acid: An integrated biomarker approach to evaluating toxicity. RSC Adv..

[46] Yang H.B., Zhao Y.Z., Tang Y., Gong H.Q., Guo F., Sun W.H., Liu S.S., Tan H., Chen F. (2019). Antioxidant defence system is responsible for the toxicological interactions of mixtures: A case study on PFOS and PFOA in *Daphnia magna*. Sci. Total. Environ..

[47] Rajak P., Ganguly A. (2023). The ligand-docking approach explores the binding affinity of PFOS and PFOA for major endogenous antioxidants: A potential mechanism to fuel oxidative stress. Sust. Chem. Environ..

[48] Mayilswami S., Krishnan K., Megharaj M., Naidu R. (2014). Chronic PFOS exposure alters the expression of neuronal development-related human homologues in *Eisenia fetida*. Ecotoxicol. Environ. Saf..

[49] Keillor J.W., Brown R.S. (1992). Attack of zwitterionic ammonium thiolates on a distorted anilide as a model for the acylation of papain by amides. A simple demonstration of a bell-shaped pH/rate profile. J. Am. Chem. Soc..

[50] Zhao Y., Li G., Qi D., Sun L., Wen C., Yin S. (2017). Biomarker responses of earthworms (*Eisenia fetida*) to soils contaminated with perfluorooctanoic acid. Environ. Sci. Pollut. Res..

[51] Briels N., Ciesielski T.M., Herzke D., Jaspers V.L.B. (2018). Developmental toxicity of perfluorooctanesulfonate (PFOS) and its chlorinated polyfluoroalkyl ether sulfonate alternative F-53B in the domestic chicken. Environ. Sci. Technol..

[52] Cerenius L., Söderhäll K. (2004). The prophenoloxidase-activating system in invertebrates. Immunol. Rev..

[53] Nappi A.J., Ottaviani E. (2000). Cytotoxicity and cytotoxic molecules in invertebrates. BioEssays.

[54] Ehrlich V., Bil W., Vandebriel R., Granum B., Luijten M., Lindeman B., Grandjean P., Kaiser A.M., Hauzenberger I., Hartmann C. (2023). Consideration of pathways for immunotoxicity of per-and polyfluoroalkyl substances (PFAS). Environ. Health.

[55] Crawford L., Halperin S.A., Dzierlenga M.W., Skidmore B., Linakis M.W., Nakagawa S., Longnecker M.P. (2023). Systematic review and meta-analysis of epidemiologic data on vaccine response in relation to exposure to five principal perfluoroalkyl substances. Environ. Int..

[56] DeWitt J.C., Blossom S.J., Schaider L.A. (2019). Exposure to per-fluoroalkyl and polyfluoroalkyl substances leads to immunotoxicity: Epidemiological and toxicological evidence. J. Expo. Sci. Environ. Epidemiol..

[57] Lionetto M.G., Caricato R., Calisi A., Giordano M.E., Schettino T. (2013). Acetylcholinesterase as biomarkers in environmental and occupational medicine: New insights and future perspectives. Biomed. Res. Int..

[58] Dondero F., Calisi A., Sebastià M.T. (2015). Evaluation of Pollution Effects in Marine Organisms: “Old” and “New Generation” Biomarkers. Coastal Ecosystems: Experiences and Recommendations for Environmental Monitoring Programs.

[59] Mulkiewicz E., Jastorff B., Składanowski A., Kleszczyński K., Stepnowski P. (2006). Evaluation of the acute toxicity of perfluorinated carboxylic acids using eukaryotic cell lines, bacteria and enzymatic assays. Environ. Toxicol. Pharmacol..

[60] Li M.H. (2008). Effects of nonionic and ionic surfactants on survival, oxidative stress, and cholinesterase activity of planarian. Chemosphere.

[61] Jeong T.Y., Yuk M.S., Jeon J., Kim S.D. (2016). Multigenerational effect of perfluorooctane sulfonate (PFOS) on the individual fitness and population growth of *Daphnia magna*. Sci. Total. Environ..

[62] Nusair S.D., Abu Zarour Y.S., id Altarifi A.A. (2017). Effects of dibenzo-p-dioxins/dibenzofurans on acetylcholinesterase activity and histopathology of the body wall of earthworm *Eisenia andrei*: A potential biomarker for ecotoxicity monitoring. Water Air Soil Pollut..

[63] He F., Liu R., Tian G., Qi Y., Wang T. (2023). Ecotoxicological evaluation of oxidative stress-mediated neurotoxic effects, genetic toxicity, behavioral disorders, and the corresponding mechanisms induced by fluorene-contaminated soil targeted to earthworm (*Eisenia fetida*) brain. Sci. Total. Environ..

[64] Brown-Leung J.M., Cannon J.R. (2022). Neurotransmission Targets of Per- and Polyfluoroalkyl Substance Neurotoxicity: Mechanisms and Potential Implications for Adverse Neurological Outcomes. Chem. Res. Toxicol..

[65] Chen N., Li J., Li D., Yang Y., He D. (2014). Chronic Exposure to Perfluorooctane Sulfonate Induces Behavior Defects and Neurotoxicity through Oxidative Dam-ages, In Vivo and In Vitro. PLoS ONE.

[66] Fu H., Xia Y., Chen Y., Xu T., Xu L., Guo Z., Xu H., Xie H.Q., Zhao B. (2018). Acetylcholinesterase is a potential biomarker for a broad spectrum of organic environmental pollutants. Environ Sci Technol..

[67] Wang N., Jagani R., Nwobodo N., Ma J. (2023). Toxicity of environmentally relevant concentration of PFAS chemicals in *Lumbriculus variegatus* (Oligochaeta, Lumbriculidae)—A multi-bioindicator study. Ecotoxicol. Environ. Saf..

[68] Gaballah S., Swank A., Sobus J.R., Howey X.M., Schmid J., Catron T., Mc Cord J., Hines E., Strynar M., Tal T. (2020). Evaluation of Developmental Toxicity, Developmental Neurotoxicity, and Tissue Dose in Zebrafish Exposed to GenX and Other PFAS. Environ. Sci. Pollut. Res..

