# Supplementary material for: Impact of Short-Chain Perfluoropropylene Oxide Acids on Biochemical and Behavioural Parameters in Eisenia fetida (Savigny, 1826)
Source: J Xenobiot. 2024 Dec 26;15(1):2. doi: 10.3390/jox15010002 (PMC11755600; doi:10.3390/jox15010002)
Supplement: Supplementary file 1 [file jox-15-00002-s001.zip › jox-3328346-supplementary.pdf]

Supplementary Materials: Impact of Short-Chain Perfluoropropylene Oxide Acids on Biochemical and Behavioural Parameters in *Eisenia fetida* (Savigny, 1826)

Davide Rotondo, Davide Gualandris, Candida Lorusso, Albert Braeuning, Antonio Calisi and Francesco Dondero

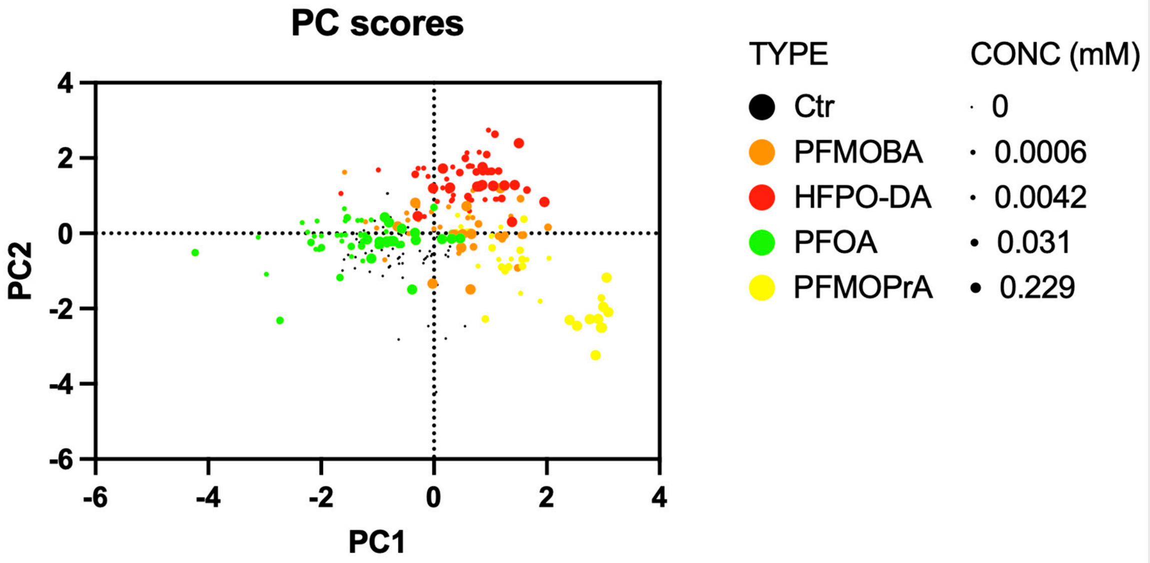

Figure S1. Principal component analysis on standardised data. The loading plot shows the segregation of treatments. PC1 and PC2 explain 35% and 25% data variance, respectively.

Expert system visualisation

Earthworm responses were classified using an expert system.

The following table (Table S1) summarises the effects found for each investigated variable and includes the PCA separation plot.

Table S1. PFAS congener potency summary.

PFAS Congen	Catalase Activity	SOD Activity	Phenol Oxidase Activity	AChE Activity	Behavioral Effects	PCA Separation
HFPO-DA (C6)	Strong, dose-dependent effects	Consistent inhibition across c	Marked inhibition at 31 μM	Strong inhibition at all do:	Significant latency delay	Distinct separation
PFMOPrA (C4)	Strong, concentration-dependent increase	No significant effect	Significant inhibition at all d	No significant effect	Variable latency delay	Moderate separation
PFMOBA (C5)	Intermediate effects at mid doses	No significant effect	Significant at highest dose	No significant effect	Minor latency effect	Moderate separation
PFOA	No significant effects	Increased at low dose only	No significant effect	Moderate inhibition at 4.2	Moderate latency reduct	Limited separation

Scoring Criteria and Results for PFAS Congeners

The potency of PFAS congeners was assessed using a transparent scoring system based on their effects across five biochemical and behavioural endpoints: catalase activity, superoxide dismutase (SOD) activity, phenol oxidase activity, acetylcholinesterase (AChE) activity, and behavioural responses. Each endpoint was scored from 1 to 5 based on the observed effects (Table S2):

Table S2. Scoring system.

Score	Effect Description
1	Minimal or no effect compared to control.
2	Mild effect, limited to specific concentrations.
3	Moderate effect with dose-specific impacts or variability across concentrations.
4	Strong effect at most concentrations, showing consistent dose-response relationships
5	Robust and consistent effect across concentrations, indicating significant toxicity

The cumulative score for each PFAS congener was calculated by summing the scores across all endpoints (Table S3). The Principal Component Analysis (PCA) plot was excluded from scoring to avoid redundancy, as it summarises variance derived from the same biochemical and behavioural data.

Table S3. Scoring system.

PFAS	Chain lenght	Catalase activity	SOD activity	Phenol oxidase	Ache activity	Behaviur	Cumulative score
HFPO-DA	C6	5	5	5	5	5	25
PFMOBA	C5	3	2	3	2	3	13
PFMOPrA	C4	4	4	5	3	4	20
PFOA	C8	2	2	1	3	2	10

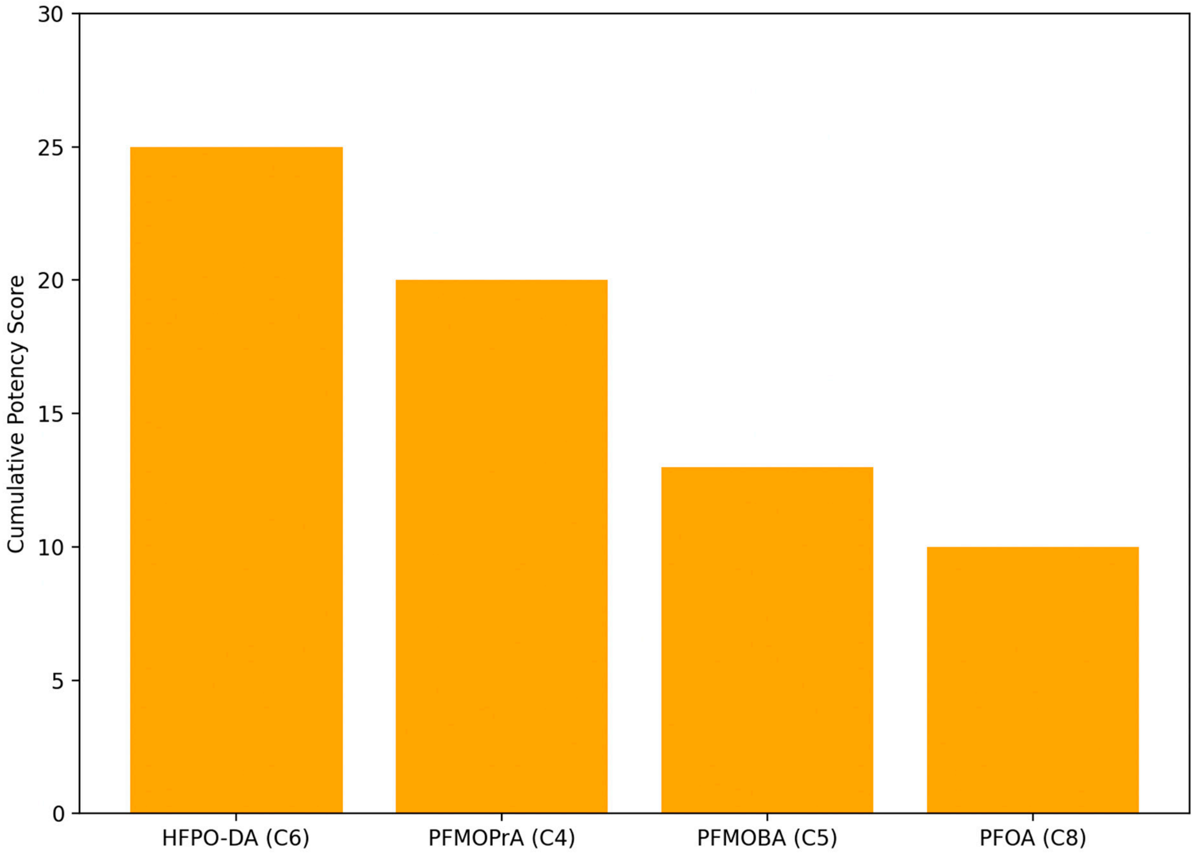

Figure S2. PFPOA potency vs. PFOA.

Interpretation

HFPO-DA (C6) demonstrated the highest potency, with strong and consistent effects across all endpoints (score: 25). This highlights its significant oxidative stress induction, immune disruption, neurotoxicity, and behavioural impact.

PFMOPrA (C4) ranked second (score: 20), showing robust effects but with slightly weaker impacts on AChE and behavioural endpoints than HFPO-DA.

PFMOBA (C5) displayed intermediate potency (score: 13), primarily affecting oxidative stress and immune responses, with limited impacts on neurotoxicity and behaviour.

PFOA, used as a reference compound, showed the least potency (score: 10), with minor effects across most endpoints.
